# Growth trajectories in exclusively breastfed infants with clinically diagnosed food allergy: a prospective longitudinal cohort study

**DOI:** 10.3389/fnut.2026.1924841

**Published:** 2026-10-23

**Authors:** Jun Fang, Zhanzhan Zhang, Yuanfeng Zhong, Yannan Wan, Qian Chen, Xuelin Xia, Xuan Zhang

**Affiliations:** 1 Department of Growth, Development and Mental Health of Children and Adolescence Center of Children’s Hospital of Chongqing Medical University, National Clinical Research Center for Children and Adolescents' Health and Diseases, Ministry of Education Key Laboratory of Child Development and Disorders. Chongqing Key Laboratory of Child Neurodevelopment and Cognitive Disorders, Chongqing, China; 2 Chongqing Key Laboratory of Child Neurodevelopment and Cognitive Disorders, Chongqing, China

**Keywords:** breastfeeding, food allergy, infant growth, maternal dietary avoidance, weight-for-length

## Abstract

**Background:**

Food allergy (FA) in infancy may be accompanied by feeding difficulties and dietary modifications during breastfeeding, which may influence early growth. However, longitudinal growth patterns in exclusively breastfed infants with clinically diagnosed FA remain incompletely characterized.

**Methods:**

In this prospective longitudinal cohort study, 73 exclusively breastfed infants (30 with FA and 43 without FA) were enrolled at 4–6 months of age and followed through infancy. A total of 268 anthropometric observations were included in the longitudinal analysis. Weight-for-length Z-score (WLZ), weight-for-age Z-score (WAZ), length-for-age Z-score (LAZ), and head circumference-for-age Z-score (HCZ) were calculated according to the WHO Child Growth Standards. Growth trajectories were evaluated using linear mixed-effects models with infant-specific random intercepts. Age was modeled continuously and centered at 5 months. Sensitivity analyses examined nonlinear age effects and group-by-sex interactions.

**Results:**

Maternal dietary avoidance was substantially more common among mothers of infants with FA than among those without FA (83.3% vs. 18.6%, *p* < 0.001). In the longitudinal WLZ model, the estimated difference between the NFA and FA groups at 5 months was 0.423 *Z*-score units (95% CI 0.036 to 0.811, *p* = 0.035), with lower estimated WLZ in the FA group. There was no evidence of a group-by-age interaction (estimate = −0.011, 95% CI − 0.086 to 0.063, *p* = 0.763), indicating no detectable difference in the linear rate of WLZ change between groups. Sensitivity analysis did not support a nonlinear WLZ trajectory, and no group-by-sex interaction was detected.

**Conclusion:**

Infants with clinically diagnosed FA had lower estimated WLZ around 5 months of age than infants without FA, but the longitudinal analysis did not provide evidence that WLZ changed at a different linear rate between groups over time. Maternal dietary avoidance was substantially more common among mothers of infants with FA, underscoring the importance of appropriate nutritional counseling and growth monitoring in this population.

## 1 Introduction

Food allergy (FA) is one of the most common childhood disorders, with increasing prevalence worldwide (1). In infancy, FA may present with eczema, gastrointestinal symptoms, feeding difficulties, and dietary restrictions, all of which may adversely affect nutritional status and growth (2). Although multiple studies have suggested an association between FA and impaired growth outcomes, particularly in children with severe or multi-food allergies-the overall evidence remains inconsistent, and the underlying mechanisms have not been fully elucidated (3, 4).

Breastfeeding is universally recommended as the optimal source of nutrition during early life, including for infants with diagnosed or suspected FA (5). In clinical practice, mothers of infants with suspected or confirmed FA frequently adopt elimination diets, either on medical advice or based on personal concerns (6). While targeted elimination of specific allergens may be clinically justified in selected cases, excessive or prolonged dietary restriction can compromise maternal nutrient intake and dietary diversity, potentially altering the nutritional composition of breast milk and indirectly influencing infant growth (7, 8). Importantly, current international guidelines do not support routine maternal dietary avoidance for allergy prevention and instead caution against unnecessary restriction during breastfeeding (9).

Despite these considerations, most previous research on growth in children with FA has focused primarily on dietary restriction applied directly to affected children, as well as on feeding difficulties and nutrient deficiencies in pediatric populations (10, 11). By contrast, relatively few studies have described maternal dietary behavior during lactation in relation to growth among exclusively breastfed infants with FA. Moreover, existing evidence is largely derived from cross-sectional analyses or mixed feeding populations, limiting the ability to characterize dynamic growth trajectories during exclusive breastfeeding (12, 13). Consequently, longitudinal growth patterns in exclusively breastfed infants with FA, together with the maternal dietary practices accompanying this condition, remain insufficiently characterized.

To address this gap, we conducted a prospective longitudinal cohort study of exclusively breastfed infants with and without FA. By focusing on a homogeneous feeding pattern during early infancy and using repeated anthropometric assessments, we aimed to characterize longitudinal growth trajectories in infants with and without FA and to describe maternal dietary avoidance practices during exclusive breastfeeding.

## 2 Materials and methods

### 2.1 Study design and participants

This was a longitudinal cohort study conducted at the Child Health Care Department of the Children’s Hospital of Chongqing Medical University, Chongqing, China. A total of 73 mother-infant pairs were enrolled between November 2024 and November 2025. This cohort focused specifically on exclusively breastfed infants with clinically diagnosed FA, a population in which longitudinal growth data remain limited. The repeated-measures design, with up to five observations per infant, provides enhanced statistical efficiency for detecting growth trajectory differences between groups. Infants were eligible if they were: (1) 4–6 months of age at enrollment; (2) exclusively breastfed; (3) full-term (gestational age ≥ 37 weeks and < 42 weeks); and (4) free of major congenital diseases. Exclusion criteria included neonatal hospitalization > 30 days, major illness or surgery during follow-up, or relocation that precluded continued follow-up. Infants with any diagnosed comorbidity that could affect growth (e.g., congenital heart disease, chronic lung disease, endocrine disorders, chromosomal abnormalities, or significant gastroesophageal reflux disease requiring medical treatment) were also excluded.

### 2.2 Diagnosis of food allergy (clinical assessment)

In this pragmatic real-world cohort, FA was diagnosed clinically based on symptom response to maternal dietary elimination and subsequent reintroduction, rather than by universal oral food challenges (OFC). This approach reflects standard clinical practice for breastfed infants—where OFC is often infeasible—and aligns with previous observational studies (14, 15).

All infants were evaluated by experienced pediatric allergists using a standardized algorithm, comprising five sequential steps: first, a detailed clinical history was obtained, assessing presenting symptoms (skin: eczema, urticaria, angioedema; gastrointestinal: vomiting, diarrhea, blood in stool, poor feeding; respiratory: wheeze, rhinitis), timing of symptom onset in relation to feeding, and family history of atopy; second, a systematic physical examination was performed to evaluate cutaneous manifestations, growth parameters, and systemic signs of allergic disease; third, for suspected cases, mothers eliminated the suspected allergen(s) from their diet for 2–4 weeks, with the specific foods eliminated based on clinical history and symptom pattern; fourth, clinical improvement following maternal elimination was recorded; and fifth, suspected foods were reintroduced one at a time over 3–5 days, with careful monitoring for symptom recurrence, where a positive response was defined as reproducible symptom recurrence within 24–72 h of reintroduction. Infants were assigned to the non-FA group if they had no history of reproducible allergic symptoms, no clinical signs of allergic disease on examination, and no evidence of allergic sensitization according to the assessing allergist. We emphasize that FA diagnoses in this cohort are clinically based and should not be equated with universally challenge-confirmed FA; throughout this manuscript, “FA” refers to this clinically defined entity.

### 2.3 Data collection and follow-up

Infants were enrolled at 4–6 months of age (before complementary feeding introduction) and followed prospectively according to routine well-child visit schedules: monthly at 4, 5, 6 months of age, and then every 2 months at 8, 10, and 12 months of age. The follow-up interval from 6 to 8 months represents a 2-month gap, as visits occurred at 6 months and then again at 8 months. At each visit, weight and length were measured by trained nurses using standardized equipment. Anthropometric measurements were converted to age- and sex-specific Z-scores, including weight-for-age Z-score (WAZ), length-for-age Z-score (LAZ), weight-for-length Z-score (WLZ), and head circumference-for-age Z-score (HCZ), using the WHO Child Growth Standards (R package Anthro) (16). At enrollment, maternal dietary avoidance practices since the infant‘s birth were documented using a structured questionnaire that captured: avoided food categories (milk, eggs, wheat, soy, nuts, fish, shellfish, peanuts), duration of avoidance and reasons for avoidance.

### 2.4 Ethics statement

This study was approved by the Ethics Committee of the Children’s Hospital of Chongqing Medical University (Approval No.: 2024 Lun Shen (Lin Yan) Pi Jian No. (419); Clinical Research Registration No.: MR-50-25-026075) and was registered in the Chinese Clinical Trial Registry (Registration No.: ChiCTR2500114005). Written informed consent was obtained from the legal guardians of the participants.

### 2.5 Statistical analysis

Longitudinal growth trajectories were analyzed using linear mixed-effects models (LMMs) with random intercepts for each infant to account for within-subject correlations arising from repeated measurements. Separate models were fitted for WLZ, WAZ, and LAZ as outcome variables. Fixed effects included group (FA vs. NFA), age (centered at 5 months), the group-by-age interaction, and infant sex as a covariate. The group-by-age interaction was used to evaluate whether longitudinal changes in growth differed between the FA and NFA groups.

For descriptive presentation, observed growth Z-scores were summarized by predefined age intervals (4 to <6, 6 to <8, 8 to <10, 10 to <12, and ≥12 months). These age-specific summaries were treated as descriptive, whereas longitudinal statistical inference was based on the mixed-effects models.

To assess the adequacy of the linear age specification, sensitivity analyses were performed by adding quadratic age terms and group-by-quadratic-age interactions to the longitudinal models. In additional sensitivity analyses, group-by-sex interaction terms were examined to assess potential effect modification by infant sex.

Model assumptions were evaluated using residual-versus-fitted plots and quantile–quantile plots of residuals. Model convergence and singularity were also examined. All statistical analyses were performed using R version 4.3.3. All tests were two-sided, and *p* < 0.05 was considered statistically significant.

## 3 Results

### 3.1 Participant characteristics

A total of 73 mother–infant pairs (30 in the FA group and 43 in the NFA group) were included, contributing 268 anthropometric observations to the longitudinal analysis (mean, 3.67 observations per infant). The number of available observations varied across age intervals and decreased at later follow-up. Group-specific sample sizes for each predefined age interval are presented in Table 1.

**Table 1:** Observed growth *Z*-scores by age interval and food allergy status.

Age interval	Outcome	NFA, n observations (n infants)	NFA mean ± SD	FA n observations (n infants)	FA mean ± SD
4 to <6 months	WLZ	46 (32)	0.61 ± 0.78	27 (22)	0.11 ± 0.96
	WAZ	46 (32)	0.28 ± 0.92	27 (22)	0.02 ± 0.99
	LAZ	46 (32)	−0.18 ± 0.99	27 (22)	0.02 ± 1.16
	HCZ	46 (32)	−0.17 ± 0.86	27 (22)	−0.31 ± 0.95
6 to <8, months	WLZ	43 (37)	0.76 ± 0.93	29 (22)	0.43 ± 0.95
	WAZ	43 (37)	0.42 ± 1.08	29 (22)	0.19 ± 1.07
	LAZ	43 (37)	−0.11 ± 1.12	29 (22)	−0.06 ± 1.36
	HCZ	43 (37)	−0.04 ± 1.05	29 (22)	−0.25 ± 0.88
8 to <10 months	WLZ	40 (33)	0.67 ± 0.88	17 (13)	0.33 ± 1.05
	WAZ	40 (33)	0.30 ± 0.93	17 (13)	0.01 ± 1.21
	LAZ	40 (33)	−0.28 ± 1.10	17 (13)	−0.31 ± 1.29
	HCZ	40 (33)	−0.05 ± 1.00	17 (13)	−0.54 ± 0.78
10 to <12 months	WLZ	30 (27)	0.43 ± 0.80	10 (10)	0.26 ± 0.81
	WAZ	30 (27)	0.25 ± 0.85	10 (10)	0.11 ± 0.89
	LAZ	30 (27)	−0.07 ± 1.22	10 (10)	−0.12 ± 1.03
	HCZ	30 (27)	−0.16 ± 0.91	10 (10)	−0.19 ± 0.97
≥12 months	WLZ	15 (14)	−0.12 ± 0.95	3 (3)	0.17 ± 0.84
	WAZ	15 (14)	−0.22 ± 1.01	3 (3)	−0.04 ± 0.93
	LAZ	15 (14)	−0.27 ± 1.21	3 (3)	−0.36 ± 0.67
	HCZ	15 (14)	−0.40 ± 0.70	3 (3)	0.60 ± 1.19

WLZ, weight-for-length *Z*-score; WAZ, weight-for-age *Z*-score; LAZ, length-for-age *Z*-score; HCZ, head circumference-for-age *Z*-score. Values are presented as mean ± SD. Sample sizes are shown as number of observations (number of unique infants). These age-specific summaries are descriptive only; longitudinal statistical inference was based on the mixed-effects models shown in Table 3. Observations obtained before 4 months of age were retained in the longitudinal dataset but were not included in these predefined descriptive age intervals.

The most common FA phenotypes in the FA group were cow’s milk protein allergy (*n* = 18, 60.0%), egg allergy (*n* = 13, 43.3%), shellfish allergy (*n* = 12, 40.0%), fish allergy (*n* = 8, 26.7%), and peanut allergy (*n* = 5, 16.7%). The distribution of FA phenotypes is shown in Supplementary Table S1. Maternal dietary avoidance was substantially more prevalent in the FA group than in the NFA group (83.3% vs. 18.6%, *p* < 0.001), with a significantly greater number of avoided foods (median 3 vs. 1, *p* = 0.003). The most avoided foods in the FA group were milk (53.3%), fish (53.3%), shellfish (50.0%), eggs (40.0%), wheat (23.3%), soy (16.7%), and peanuts (10.0%). Reasons for maternal dietary avoidance differed substantially between groups. In the FA group (*n* = 25, 83.3%), all cases of dietary avoidance were initiated due to suspected or confirmed infant FA. Of these, 20 (80%) were physician-recommended, and 5 (20%) were initiated by mothers based on personal concerns (with subsequent physician confirmation). In the NFA group (*n* = 8, 18.6%), avoidance was primarily driven by maternal personal factors: maternal allergy/intolerance (*n* = 2, 25%), and concern about infant colic (*n* = 6, 75%). None of the NFA group mothers reported avoidance due to physician recommendation for infant FA. Most baseline characteristics were similar between groups, with the exception of maternal dietary avoidance practices. Breastfeeding duration was comparable between groups (Table 2). Detailed feeding frequency and duration per feed were not systematically recorded in this study. No infants in either group had major comorbidities that could independently affect growth, as per the exclusion criteria.

**Table 2:** Baseline characteristics and maternal dietary practices of the study population.

Characteristic	NFA group (*n* = 43)	FA group (*n* = 30)	*p*-value
Male sex, *n* (%)	24 (55.8)	15 (50.0)	0.642
Age at enrollment (days)	151.4 ± 22.1	160.0 ± 24.6	0.111
Birth weight (kg)	3.22 ± 0.36	3.17 ± 0.39	0.844
Birth length (cm)	49.8 ± 1.8	49.1 ± 1.6	0.195
Gestational age (weeks)	39.1 ± 0.9	39.2 ± 1.0	0.653
Maternal age (years)	30.1 ± 3.7	30.7 ± 3.9	0.281
Maternal pre-pregnancy BMI (kg/m^2^)	21.0 ± 3.9	20.8 ± 2.6	0.766
Family history of allergy, *n* (%)	19 (44.2)	20 (66.7)	0.094
Maternal education, *n* (%)			0.500
Bachelor or above	36 (83.7)	23 (76.7)	
Below bachelor	7 (16.3)	7 (23.3)	
Paternal education, *n* (%)			1.000
Bachelor or above	33 (76.7)	23 (76.7)	
Below bachelor	10 (23.3)	7 (23.3)	
Monthly household income (RMB), *n* (%)			0.889
>10,000	29 (67.4)	20 (66.7)	
8,000–10,000	7 (16.3)	6 (20.0)	
5,000–8,000	7 (16.3)	4 (13.3)	
<5,000	0 (0.0)	0 (0.0)	
Duration of exclusive breastfeeding (days)	142.9 ± 27.1	131.7 ± 49.6	0.267
Maternal dietary avoidance, *n* (%)	8 (18.6)	25 (83.3)	**< 0.001**
Number of avoided foods, median (Q1, Q3)	1 (0, 2)	3 (2, 5)	**0.003**
Duration of dietary avoidance (days), median (Q1, Q3)	0 (0, 0)	60 (15, 150)	**<0.001**

FA, food allergy; NFA, non-food allergy; BMI, body mass index; RMB, Renminbi (Chinese currency); Q1, first quartile; Q3, third quartile. Bold values indicate statistically significant differences (*p* < 0.05). Duration of dietary avoidance was compared using the Mann–Whitney *U*-test (*W* = 319, *p* < 0.001). Percentages may not sum to 100% due to rounding.

### 3.2 Maternal dietary avoidance patterns

Maternal dietary avoidance was markedly more common among mothers of infants with FA than among those without FA. The crude odds ratio for dietary avoidance in the FA group was 16.50(95% CI 5.01–54.38). This association remained highly significant after adjustment for maternal age (continuous), maternal education level (categorical: bachelor or above vs. below bachelor), and family history of allergy (binary) (adjusted OR 14.14, 95% CI 4.04–49.57, *p* < 0.001), indicating that maternal avoidance practices were strongly associated with the presence of infant FA.

Mothers of infants with FA avoided a significantly greater number of food categories than mothers in the NFA group (median 3 vs. 1, *p* = 0.003).

### 3.3 Descriptive growth measures across age intervals

Observed growth *Z*-scores according to the predefined age intervals are summarized in Table 1. The descriptive values showed that mean WLZ was lower in the FA group than in the NFA group during the earlier age intervals, whereas group differences were smaller at later ages. However, these age-specific summaries were not used for inferential testing, and longitudinal group differences were evaluated using the mixed-effects models described below. The number of available observations decreased at later follow-up, particularly in the FA group, and estimates at later ages should therefore be interpreted cautiously.

### 3.4 Longitudinal growth trajectories

Linear mixed-effects models were used to evaluate longitudinal growth trajectories using all available repeated anthropometric measurements (Table 3).

**Table 3:** Linear mixed-effects model results for longitudinal growth *Z*-scores.

Outcome	Fixed effect	Estimate	SE	95% CI	*p*-value
WLZ	Intercept	0.193	0.179	−0.157 to 0.543	0.283
	Group (NFA vs. FA)	0.423	0.198	0.036 to 0.811	0.035
	Age centered at 5 months	−0.054	0.033	−0.119 to 0.011	0.105
	Sex (female vs. male)	0.331	0.177	−0.017 to 0.678	0.067
	Group × Age	−0.011	0.038	−0.086 to 0.063	0.763
WAZ	Intercept	0.143	0.209	−0.265 to 0.552	0.493
	Group (NFA vs. FA)	−0.102	0.231	−0.555 to 0.351	0.659
	Age centered at 5 months	−0.197	0.041	−0.276 to −0.117	<0.001
	Sex (female vs. male)	0.861	0.205	0.458 to 1.263	<0.001
	Group × Age	0.156	0.046	0.065 to 0.247	<0.001
LAZ	Intercept	−0.105	0.217	−0.529 to 0.320	0.629
	Group (NFA vs. FA)	−0.526	0.240	−0.996 to −0.056	0.030
	Age centered at 5 months	−0.202	0.042	−0.284 to −0.120	<0.001
	Sex (female vs. male)	1.124	0.213	0.706 to 1.542	<0.001
	Group × Age	0.202	0.048	0.109 to 0.296	<0.001

WLZ, weight-for-length *Z*-score; WAZ, weight-for-age *Z*-score; LAZ, length-for-age *Z*-score; FA, food allergy; NFA, non-food allergy. Age was centered at 5 months; therefore, the group coefficient represents the estimated between-group difference at 5 months. All models included infant sex as a covariate and a random intercept for each infant. Estimates were obtained using maximum likelihood, with Satterthwaite degrees of freedom for hypothesis testing and Wald 95% confidence intervals. Quadratic sensitivity analyses indicated nonlinear age patterns for WAZ and LAZ; therefore, the corresponding linear interaction estimates should be interpreted cautiously.

For WLZ, the estimated difference between the NFA and FA groups at the centered age of 5 months was 0.423 *Z*-score units (95% CI 0.036 to 0.811, *p* = 0.035), with lower estimated WLZ in the FA group. The age effect was −0.054 *Z*-score units per month (95% CI − 0.119 to 0.011, *p* = 0.105). There was no evidence of a group-by-age interaction (estimate = −0.011, 95% CI − 0.086 to 0.063, *p* = 0.763), indicating that the linear rate of WLZ change did not differ significantly between groups.

For WAZ and LAZ, significant linear group-by-age interactions were observed (WAZ: estimate = 0.156, 95% CI 0.065 to 0.247, *p* < 0.001; LAZ: estimate = 0.202, 95% CI 0.109 to 0.296, *p* < 0.001). However, quadratic sensitivity analyses indicated significant nonlinear age patterns for both outcomes; therefore, these linear interaction estimates were interpreted cautiously rather than as evidence of constant linear divergence over time.

Sensitivity analysis did not support a nonlinear WLZ trajectory: adding quadratic age terms did not significantly improve model fit (*p* = 0.192), and the group-by-quadratic-age interaction was not significant (*p* = 0.656). Additional sensitivity analyses showed no evidence of group-by-sex interactions for WLZ (*p* = 0.599), WAZ (*p* = 0.980), or LAZ (*p* = 0.825).

Model-predicted WLZ trajectories with 95% confidence intervals and observed age-specific group means are shown in Figure 1. Quadratic WAZ and LAZ trajectories are provided in Supplementary Figure S1.

**Figure 1:**
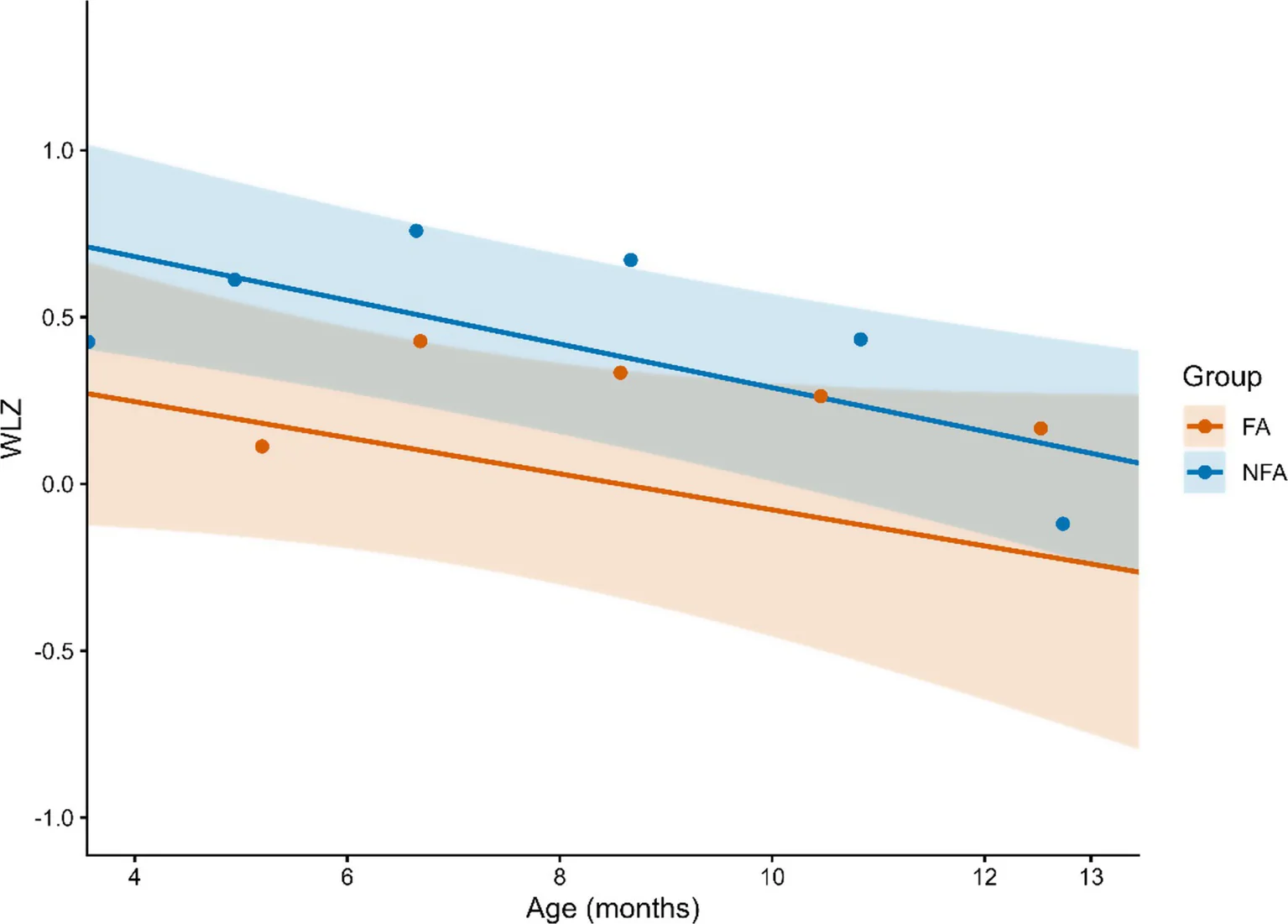
Longitudinal WLZ trajectories in infants with and without food allergy. Model-predicted weight-for-length *Z*-score (WLZ) trajectories are shown for infants with food allergy (FA) and those without food allergy (NFA), with 95% confidence intervals. Observed age-specific group means are superimposed for descriptive reference. Estimates were derived from the linear mixed-effects model including group, age centered at 5 months, the group-by-age interaction, and infant sex as fixed effects, with a random intercept for each infant. For visual clarity, the *x*-axis displays the age range from 4 to 13 months; the underlying mixed-effects model was fitted using all available longitudinal observations.

## 4 Discussion

In this prospective longitudinal cohort of exclusively breastfed infants, infants with clinically diagnosed FA had lower estimated WLZ around 5 months of age than infants without FA. This finding is broadly consistent with previous studies reporting increased vulnerability to growth impairment among children with FA, particularly those with gastrointestinal manifestations, feeding difficulties, or extensive dietary restrictions (14, 17, 18). Maternal dietary avoidance was also substantially more common among mothers of infants with FA, consistent with the frequent use of dietary modification in the clinical management of suspected or confirmed infant FA (19–21). However, in the present longitudinal analysis, there was no evidence that the linear rate of WLZ change differed between groups over time. A WLZ of zero represents the median of the WHO reference population rather than the expected mean of every study sample; therefore, positive mean WLZ values in this cohort do not themselves indicate abnormal growth (22).

By restricting the cohort to exclusively breastfed infants and using repeated anthropometric assessments, our study provides a focused view of early growth patterns and accompanying maternal dietary practices.

Maternal dietary avoidance may have nutritional implications through several pathways. Extensive avoidance of milk, eggs, wheat, fish, or other major food groups may reduce maternal dietary diversity and nutritional adequacy, particularly when such restriction occurs without professional nutritional counseling (23, 24). Maternal dietary avoidance may also reflect the clinical burden of infant symptoms, because mothers of more symptomatic infants may be more likely to adopt restrictive diets (18). In the present cohort, maternal dietary avoidance was substantially more frequent among mothers of infants with FA; however, because the revised analysis does not establish an independent association between maternal avoidance and infant growth, these mechanisms should be regarded as clinically plausible considerations rather than explanations directly demonstrated by our data. Complementary feeding may nevertheless be relevant to subsequent growth trajectories in infants with FA. As complementary foods are introduced, dietary diversity and nutrient sources increase beyond breast milk alone, which may influence weight gain and body proportionality during later infancy. However, detailed information on the timing, composition, quantity, and nutrient density of complementary foods was not collected in this cohort. Therefore, the present data cannot determine whether complementary feeding contributed to the later growth pattern observed in either group. Future prospective studies incorporating detailed complementary feeding records will be needed to assess whether dietary diversification modifies growth trajectories in infants with FA. The longitudinal patterns of WAZ and LAZ differed from that of WLZ. In the primary linear models, significant group-by-age interactions were observed for both WAZ and LAZ. However, quadratic sensitivity analyses indicated significant nonlinear age patterns for these outcomes, suggesting that their trajectories were not adequately summarized by a constant linear slope over time. These findings should therefore be interpreted cautiously and as evidence of more complex age-related growth patterns rather than simple linear divergence between the FA and NFA groups. By contrast, the WLZ trajectory was adequately described by the linear model, and no significant group-by-age interaction was detected. WLZ is a standardized measure of weight relative to length and is commonly used to assess body proportionality in infancy (22).

## 5 Implications and limitations

These findings support close growth monitoring in exclusively breastfed infants with FA. Maternal dietary avoidance was common among mothers of infants with FA, and when dietary restriction is clinically necessary, individualized nutritional counseling may help maintain maternal dietary adequacy and avoid unnecessary restriction.

This study has several strengths, including its prospective longitudinal design, repeated anthropometric measurements, focus on exclusively breastfed infants, and use of mixed-effects models to account for within-infant correlation. Several limitations should also be acknowledged. First, the sample size was modest, particularly at later follow-up ages, which limited the precision of age-specific descriptive estimates and secondary analyses. Second, detailed information on complementary feeding practices and nutrient intake was unavailable, precluding direct evaluation of their contribution to later growth patterns. Third, maternal dietary avoidance was assessed retrospectively at enrollment and detailed maternal energy, protein, and micronutrient intake were not measured, limiting interpretation of its nutritional implications. Fourth, because oral food challenges were not systematically performed, FA diagnosis was based primarily on clinical assessment and response to elimination and reintroduction, and IgE- and non-IgE-mediated phenotypes could not be distinguished. Fifth, follow-up attendance varied across age intervals, and the number of available observations decreased at later ages, particularly in the FA group. Mixed-effects models accommodated unequal numbers of repeated observations across infants, although differential follow-up may still have affected the precision of estimates at later ages. Finally, the observational, single-center design limits causal inference and generalizability to other populations and clinical settings. Larger prospective studies with detailed maternal and complementary feeding assessments are needed to clarify the relationships among FA, maternal dietary practices, and infant growth.

## 6 Conclusion

Infants with clinically diagnosed FA had lower estimated WLZ around 5 months of age than infants without FA, but no significant difference in the linear rate of WLZ change was detected between groups over time. Maternal dietary avoidance was markedly more common in the FA group. These findings support close growth monitoring and appropriate nutritional counseling for breastfed infants with FA and their mothers. Further prospective studies with detailed dietary assessment are needed to clarify the links among FA, maternal dietary practices, and infant growth.

## Data Availability

The raw data supporting the conclusions of this article will be made available by the authors, without undue reservation.
