# Supplementary material for: Growth trajectories in exclusively breastfed infants with clinically diagnosed food allergy: a prospective longitudinal cohort study
Source: Front Nutr. 2026 Oct 23;13:1924841. doi: 10.3389/fnut.2026.1924841 (PMC13739592; doi:10.3389/fnut.2026.1924841)
Supplement: Supplementary file 1 [file Supplementary_file_1.docx]

**Supplementary Table S1. Distribution of food allergy phenotypes in the FA group (n=30)**

| FA phenotype | n (%) |
| --- | --- |
| Cow's milk protein allergy | 18 (60.0) |
| Egg allergy | 13 (43.3) |
| Shellfish allergy | 12 (40.0) |
| Fish allergy | 8 (26.7) |
| Peanut allergy | 5 (16.7) |
| Soy allergy | 4 (13.3) |
| Wheat allergy | 2 (6.7) |

Note: Percentages sum to >100% because some infants had multiple food allergies.

**Supplementary Figure S1**. **Quadratic longitudinal trajectories of WAZ and LAZ in infants with and without food allergy.** Model-predicted trajectories are shown for infants with food allergy (FA) and those without food allergy (NFA), with shaded areas indicating 95% confidence intervals. Points represent observed age-specific group means for descriptive reference. Quadratic age terms were included as sensitivity analyses to assess potential nonlinearity in longitudinal growth patterns. For visual clarity, the figure displays the age range from 4 to 13 months; the underlying models were fitted using all available longitudinal observations.
